# Takotsubo cardiomyopathy following pacemaker insertion complicated with polymorphic ventricular tachycardia: a case report

**DOI:** 10.1186/s13256-024-04565-5

**Published:** 2024-05-06

**Authors:** Damanpreet Dev, Mohammed El-Din, Siddharth Vijayakumar, Rayno Navinan Mitrakrishnan

**Affiliations:** 1 Department of Cardiology, Kettering General Hospital, NHS, Kettering, UK; 2 University Hospital-Kotelawala Defence University, Colombo, Sri Lanka

**Keywords:** Takotsubo cardiomyopathy, Pacemaker induced, Polymorphic ventricular tachycardia

## Abstract

**Background:**

Takotsubo cardiomyopathy is a novel form of rapidly reversible heart failure occurring secondary to a stressor that mimics an acute coronary event. The underlying etiology of the stressor is highly variable and can include medical procedures. Pacemaker insertion is an infrequent cause of Takotsubo cardiomyopathy.

**Case presentation:**

An 86-year-old Caucasian woman underwent an uncomplicated pacemaker insertion for symptomatic complete heart block in the background of slow atrial fibrillation. A transient episode of polymorphic ventricular tachycardia was noted on day 1 following the procedure; however, her pacemaker was checked and, as she remained stable, she was discharged home. She presented again 5 days later with symptomatic heart failure. Chest X-ray confirmed pulmonary edema. Echocardiography confirmed new onset severe left ventricle dysfunction. Pacemaker checks were normal and lead placement was confirmed. Though her troponin I was elevated, her coronary angiogram was normal. Contrast enhanced echocardiography suggested apical ballooning favoring Takotsubo cardiomyopathy. She was treated for heart failure and made a good recovery. Her follow-up echocardiography a month later showed significant improvement in left ventricle function.

**Conclusions:**

Takotsubo cardiomyopathy is mediated by a neuro-cardiogenic mechanism due to hypothalamic–pituitary–adrenal axis activation. It generally has a good prognosis. Complications though uncommon, can occur and include arrhythmias. Pacemaker insertion as a precipitant stressor is an infrequent cause of Takotsubo cardiomyopathy. As pacemaker insertions are more frequent in the elderly age group, this phenomenon should be recognized as a potential complication.

**Supplementary Information:**

The online version contains supplementary material available at 10.1186/s13256-024-04565-5.

## Background

Takotsubo cardiomyopathy (TC) is a novel form of reversible heart failure mimicking an acute coronary event. It is commonly seen in postmenopausal women following emotional or physical stress [1]. Different diagnostic criteria’s try to define TC; the common criterion include transient left ventricle (LV) dysfunction that involves more than one epicardial vessel supply, new electrocardiographic changes favoring ischemia, positive cardiac biomarkers, absence of coronary vessel abnormality or systemic insult, a stressful trigger, and recovery of LV function over time [2]. TC has known to occur following various stressors, for example, infections, burn injuries, stroke, surgical procedures, and induction of anesthesia [3]. Pacemaker (PM) insertion is a minimally invasive cardiac procedure; however, it can give rise to a wide spectrum of complications, with an incidence up to 5% [4]. PM insertion leading to TC is rare. There are only a handful of case reports of this phenomenon [5–7]. In this case report, we describe a patient who underwent a single-chamber PM insertion who developed TC complicated with runs of ventricular tachycardia.

## Case presentation

An 86-year-old Caucasian female presented with presyncope. She had type 2 diabetes mellitus, hypertension, and atrial fibrillation (AF). Her routine medications were ramipril 2.5 mg once daily (OD), bisoprolol 1.25 mg OD, metformin 500 mg twice daily (BD), and apixaban 2.5 mg BD. Her blood pressure was 180/97 mmHg with a bradycardia of 32 beats per minute (bpm). The rest of her cardiac and systemic physical examination was unremarkable. Electrocardiograph (ECG) revealed complete heart block alternating with slow AF and ectopics with a variable heart rate between 29 and 33 bpm. Her investigations including thyroid functions and electrolytes were normal. She was admitted to the Coronary Care Unit (CCU) with a plan for in patient PM insertion. Her preliminary transthoracic echocardiogram (TTE) showed a mildly dilated LV with a normal ejection fraction.

**Fig. 1:**
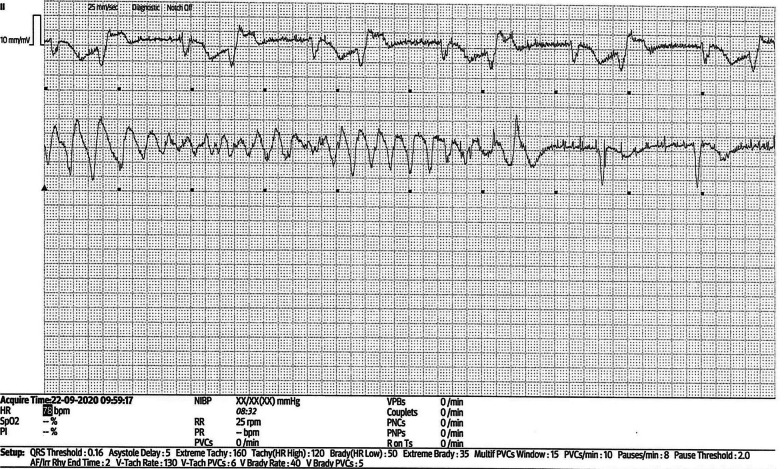
Interrogation of the permanent pace maker following the episode of collapse within the Coronary Care Unit showed runs of polymorphic ventricular tachycardia

She underwent a single-chamber VVI PM insertion. Post pacemaker chest X-ray and pacing checks were normal. On the same day, the patient felt unwell and dizzy but she attributed it to her anxiousness. On the subsequent day, she had an episode of collapse in the CCU. Interrogation of the PM revealed runs of polymorphic ventricular tachycardia (VT) (Fig. 1). The initial ventricular rate, which was set for 35–40 bpm was increased to 90 bpm, to counter the rate-related occurrence of polymorphic VT. She remained well thereafter and was discharged the following day.

**Fig. 2:**
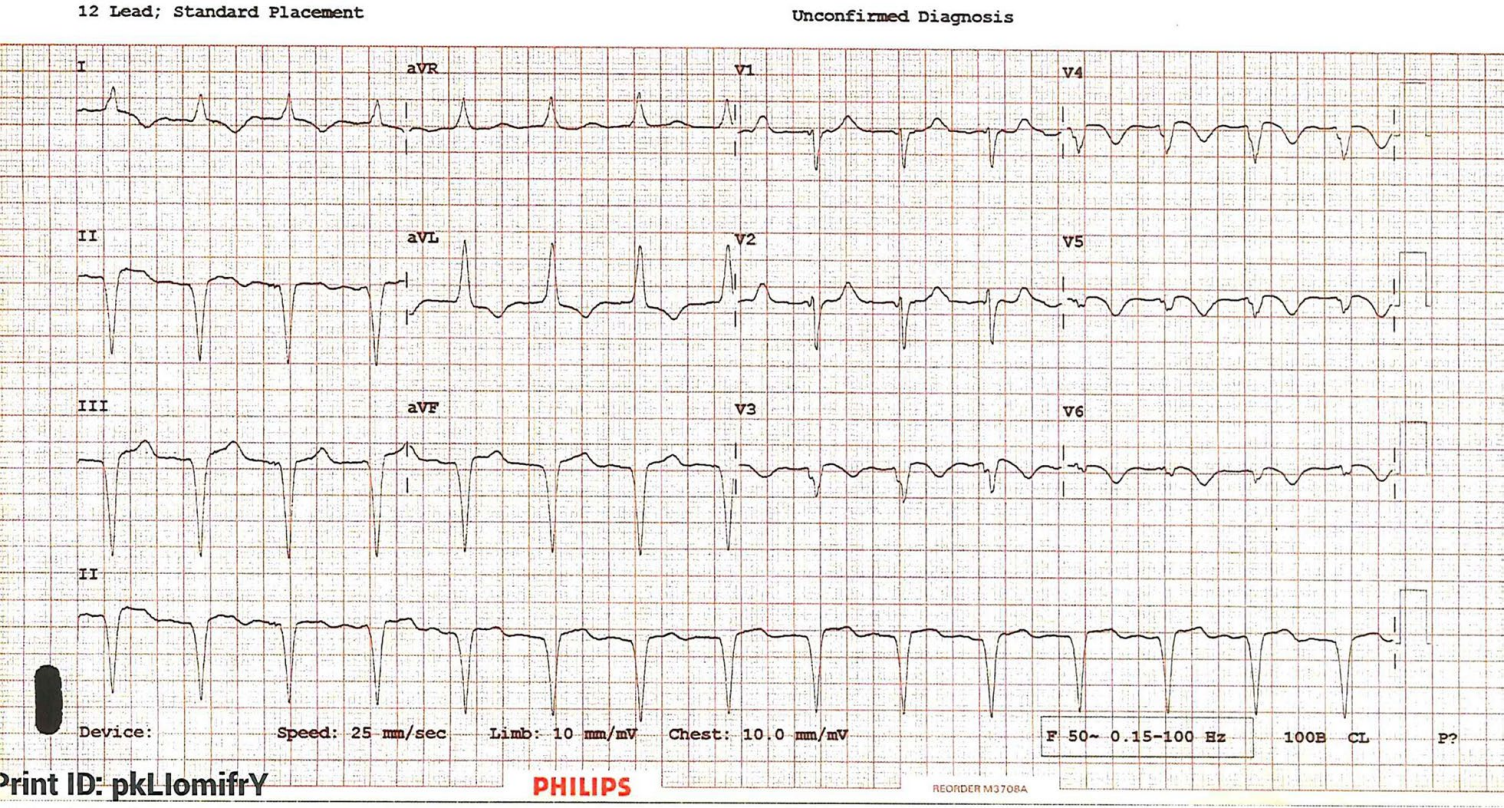
Electrocardiograph taken on readmission shows regular pacing rhythm with no other concerning features

**Fig. 3:**
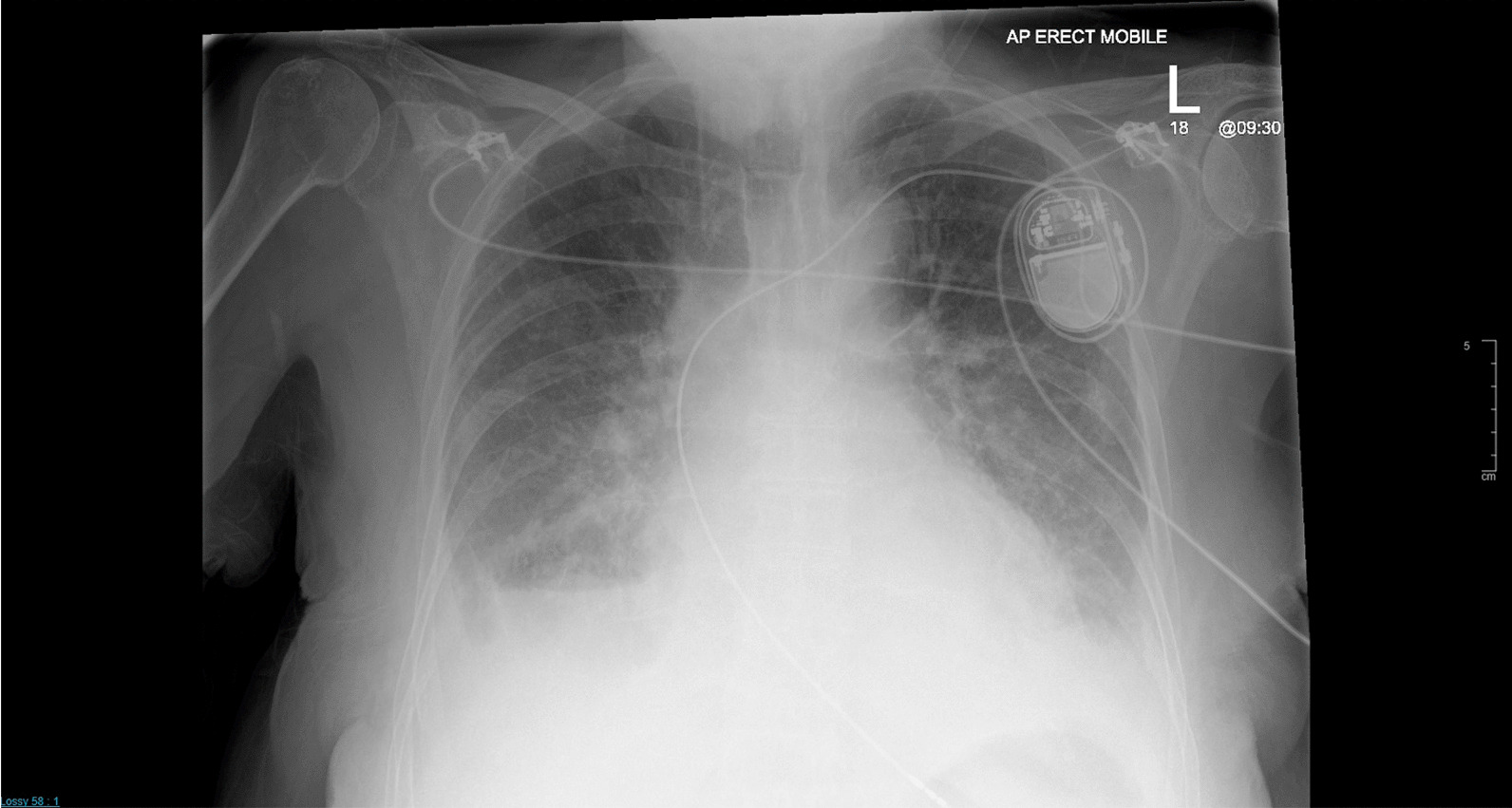
Chest X-ray taken in posteroanterior view following readmission with shortness of breath demonstrates cardiomegaly and features keeping in with heart failure

**Fig. 4:**
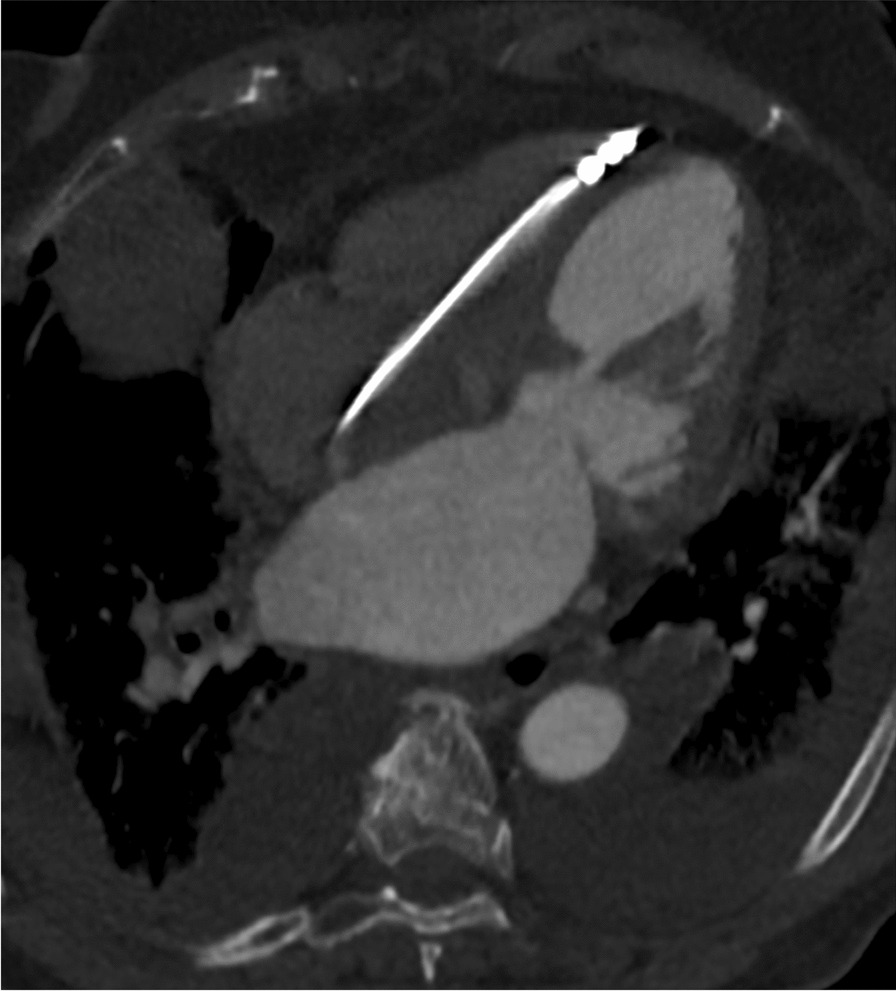
Noncontrast computed tomography scan of the chest confirmed the position of the lead of the pacemaker in the right ventricle

Five days post discharge, she presented again acutely unwell, with new onset of worsening breathlessness. Her clinical examination revealed a heart rate of 90 bpm with a blood pressure of 165/90 mmHg and a respiratory rate of 43 per minute with mild pedal edema. Auscultation of her lungs revealed bilateral mid to lower zone crackles and crepitation’s favoring pulmonary edema. The rest of her systemic examination was normal. Her on air oxygen saturation was 85%. Her high-sensitivity troponin was elevated at 90.7 ng/L. The rest of her investigations were normal. Her ECG showed a pacing rhythm (Fig. 2) and her chest X-ray demonstrated pulmonary edema, pleural effusion, and cardiomegaly. As the pacing lead position was doubtful (Fig. 3) a noncontrast ECG gated computed tomography (CT) chest was done, which ruled out perforation and confirmed the PM lead position (Fig. 4). Her PM interrogation showed 100% ventricular pacing with normal parameters and no arrhythmias. Her TTE revealed a severely impaired LV function with akinetic apex and apical ballooning. She underwent a coronary angiogram, which demonstrated normal epicardial vessels. TTE with contrast enhancement confirmed the classical features keeping in with Takotsubo cardiomyopathy (Additional file 1).

She was offloaded with intravenous furosemide and she required oxygen at 3 L/minute to maintain her saturation. Following stabilization she was initiated on heart failure medication with sacubitril/valsartan 24/26 mg BD, eplerenone 12.5 mg OD, bisoprolol 1.25 mg OD, bumetanide 1 mg OD, and her apixaban 2.5 mg BD was continued. She was discharged on day 7 with a planned follow-up with the heart failure team and the pacing team. A repeat TTE 1 month later revealed improved cardiac functionality with with an ejection fraction (EF) of 45%. There was also resolution of the apical ballooning and akinesis noted previously (Additional file 2).

## Discussion and conclusions

In most instances of TC, there is always a preceding stressor, which could be emotional or physical. The physiological response to stress results in the hypothalamic–pituitary–adrenal axis activation. This results in direct release of norepinephrine via the sympathetic nerves into the myocardium. Elevated levels of catecholamines are also seen in the circulation due to the adrenal gland activation. This causes an elevated heart rate and contractility, resulting in a mismatch between oxygen supply and demand, leading to myocyte hypoxia together with electrolyte imbalance, culminating in myocardial dysfunction. An alternate theory is that of direct catecholamine-induced myocardial toxicity [8]. Wei *et al*., in their documented event of TC following PM insertion suggested that their patient felt pain and anxiety during the procedure and attributed it as the stressor [6]. However, even after adequate analgesia and sedation, TC has been observed following PM insertion [9] suggesting that even an uncomplicated PM insertion is enough of a stressor to trigger the neuro-cardiac axis to cause TC [10]. In hindsight, our patient also had anxiety and apprehension regarding her PM insertion and this could very well have been the nidus for her to develop TC.

Female gender and advanced age is closely associated with the occurrence of TC. In most of the documented cases, the patient in concern is a postmenopausal female, a common demography at risk for TC and this remains true even for PM-induced TC [6, 9]. Estrogen receptors and beta-adreno receptors have a collaborative role in modulating cardiac physiology [11]. Estrogen down regulates cardiac adreno-receptors conferring a protective role. Following menopause low, estrogen states may lead to increased adreno-receptor levels and their activation predisposes the myocardium to adrenergic response and cardiac insult [9].

TC generally has a good prognosis, with most cases showing resolution within a few weeks to a month. However complications can occur with heart failure and cardiogenic shock, arrhythmias, LV thrombus, and rarely death [12]. Ventricular fibrillation (VT) following TC occurs due to re-entry, triggered activity, and automaticity. Its presence in TC favors a poor outcome. Comparatively, polymorphic VT are less sinister than monomorphic VT. It is hypothesized to be due to triggered activity secondary to catecholamine excess affecting intracellular calcium, resulting in delayed after depolarization [13]. PM-induced polymorphic VT is an alternate scenario, it is usually in the context of the pacing algorithm being set at a lower rate, causing QT prolongation resulting in polymorphic VT [14].

Our case report highlights a rare complication following PM insertion with TC complicated with polymorphic VT.

### Supplementary Information

**Additional file 1. **Transthoracic echocardiogram in 4 chamber view with contrast enhancement shows classical apical ballooning keeping in Takotsubo cardiomyopathy along with severe LV dysfunction.

**Additional file 2. **Transthoracic echocardiogram in 4 chamber view 1 month following discharge shows remarkable normalisation of the LV cavity and improved LV function.

## Data Availability

Data sharing is not applicable to this article as no datasets were generated or analysed during the current study.
